# Supplementary material for: Chloroplast Thylakoidal Ascorbate Peroxidase, PtotAPX, Has Enhanced Resistance to Oxidative Stress in Populus tomentosa
Source: Int J Mol Sci. 2022 Mar 19;23(6):3340. doi: 10.3390/ijms23063340 (PMC8953715; doi:10.3390/ijms23063340)

Figure 1D

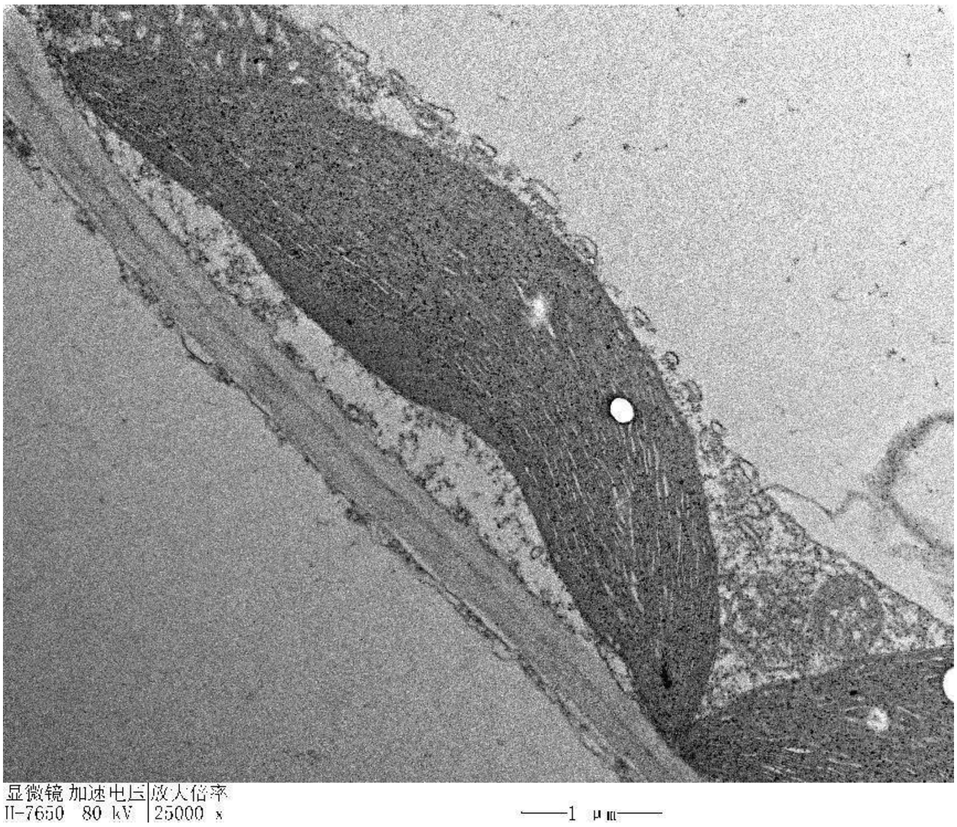

Figure 1E

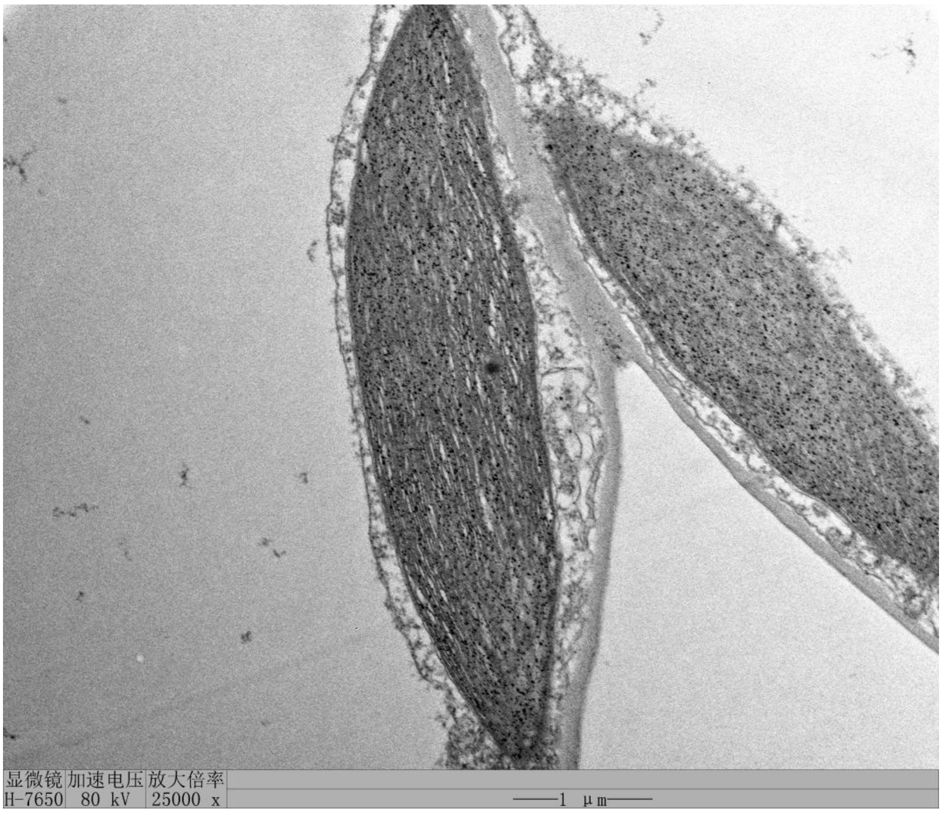

Figure 1F

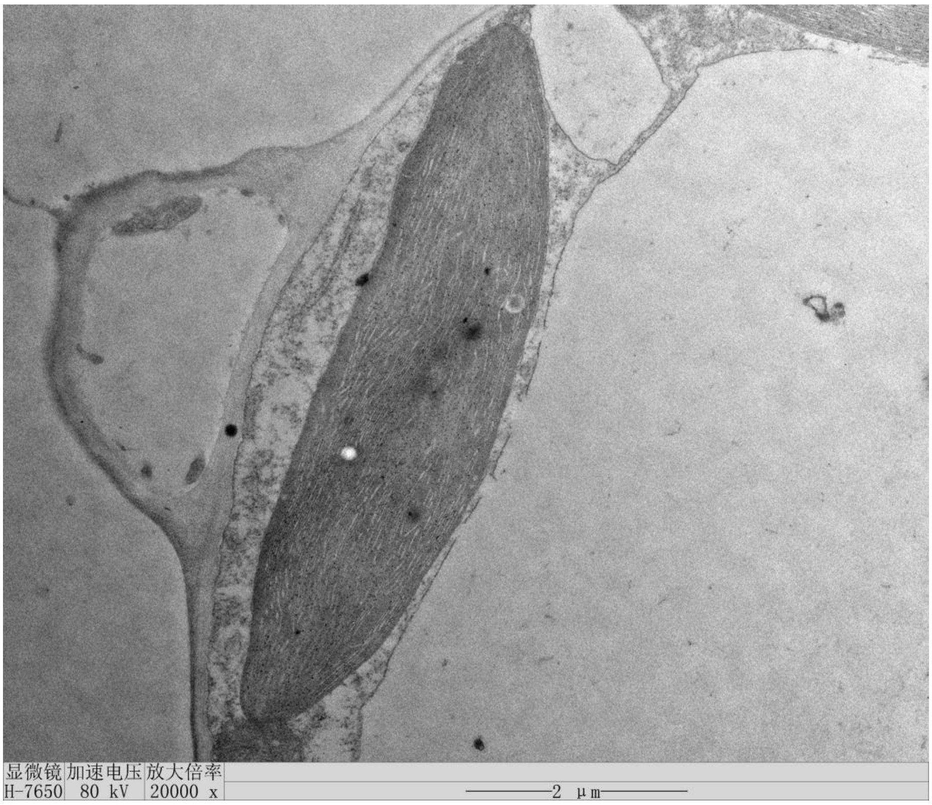

Figure 2A

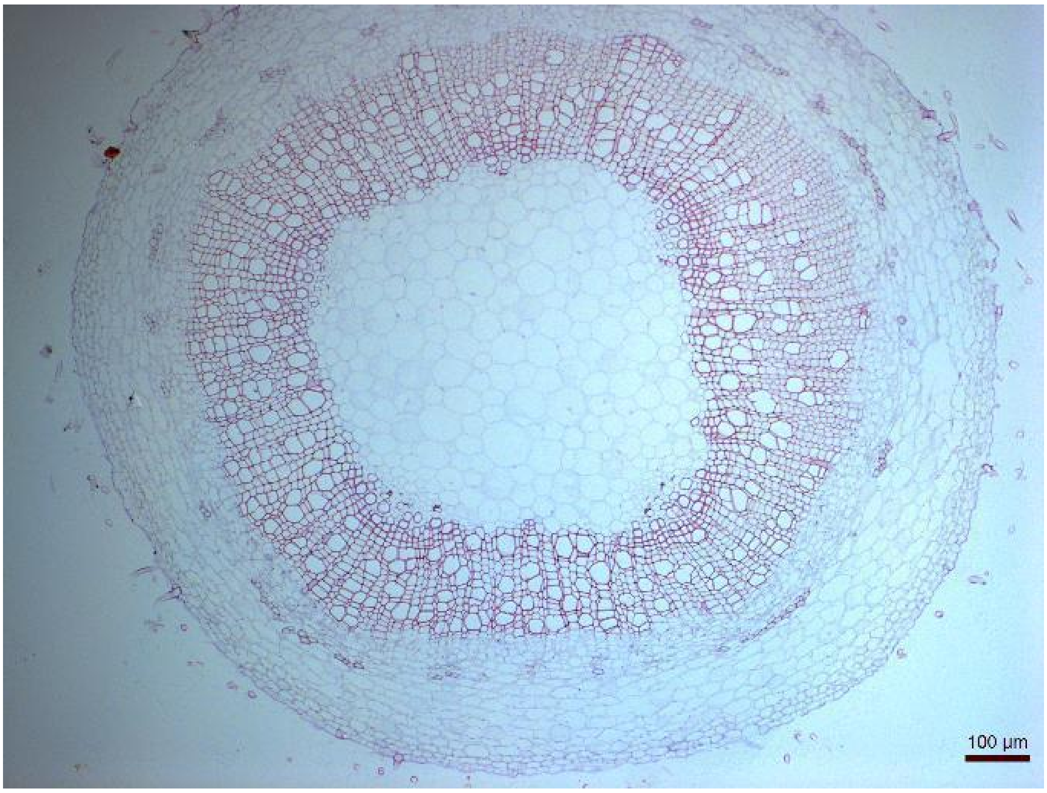

Figure 2B

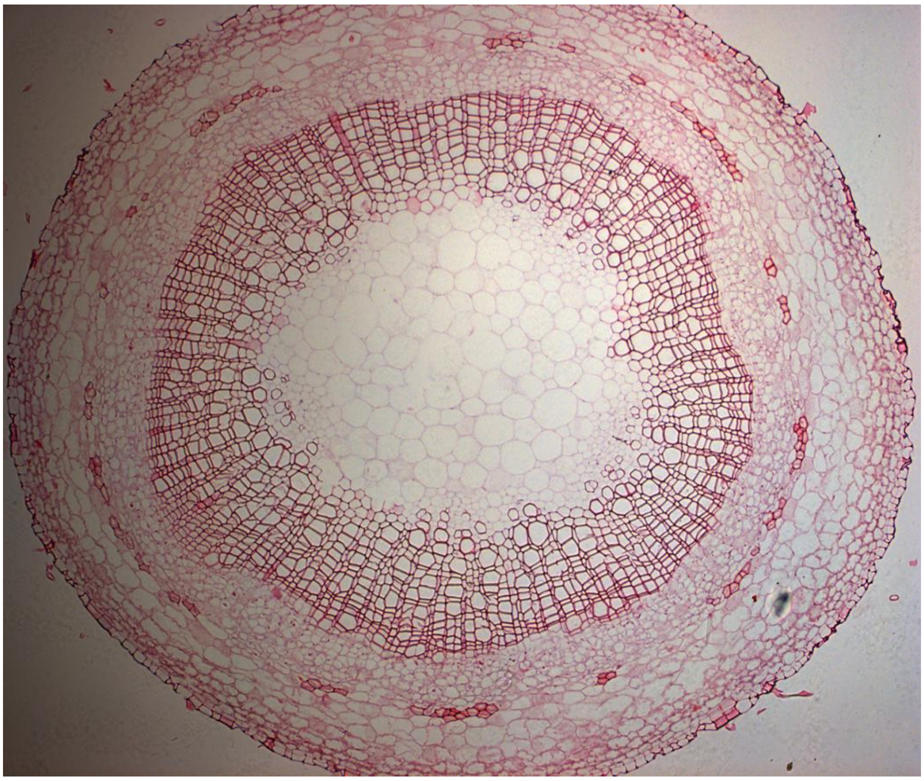

Figure 2C

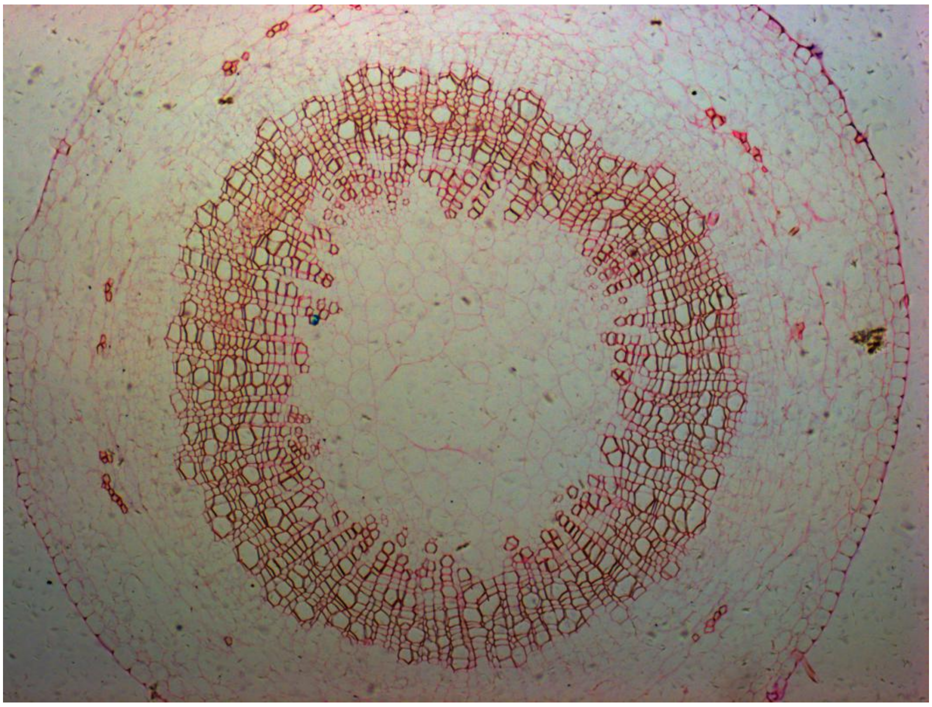

Figure 2D

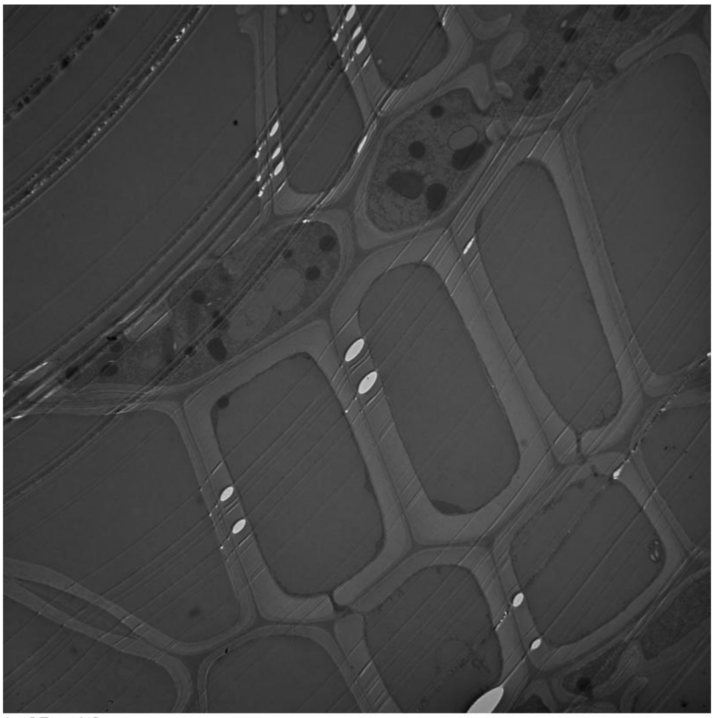

1-37.tif
Print Mag: 4750x @ 7.0 in
TEM Mode: Imaging

10 µm
HV=79.0kV
Direct Mag: 4000x
Tilt:
AMT Camera System

Figure 2E

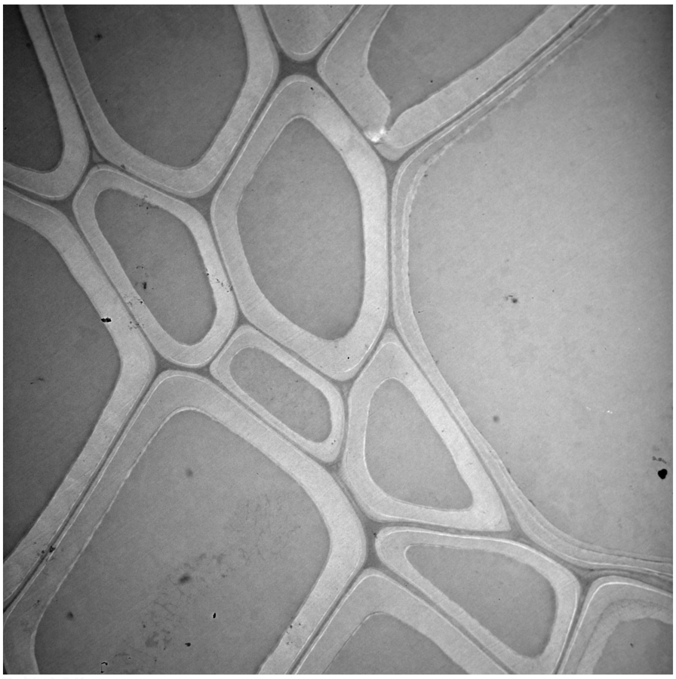

3-7.tif
Print Mag: 5940x @ 7.0 in
TEM Mode: Imaging

2 µm
HV=79.0kV
Direct Mag: 5000x
Tilt:
AMT Camera System

Figure 2F

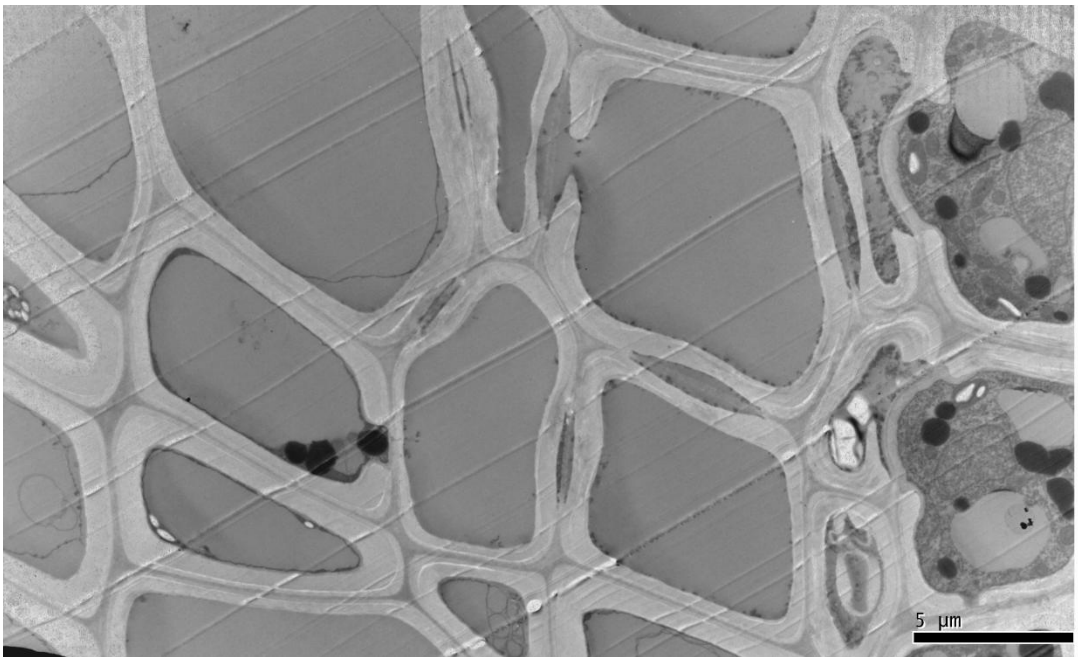

Figure 3D

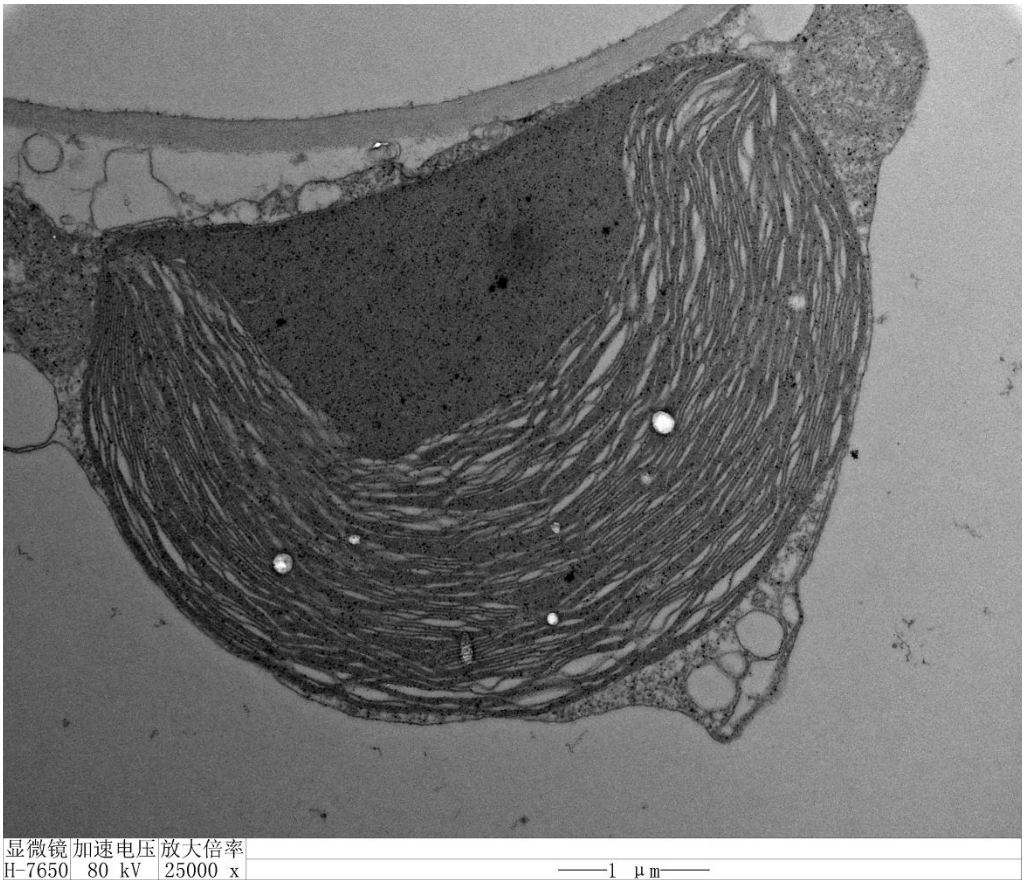

Figure 3E

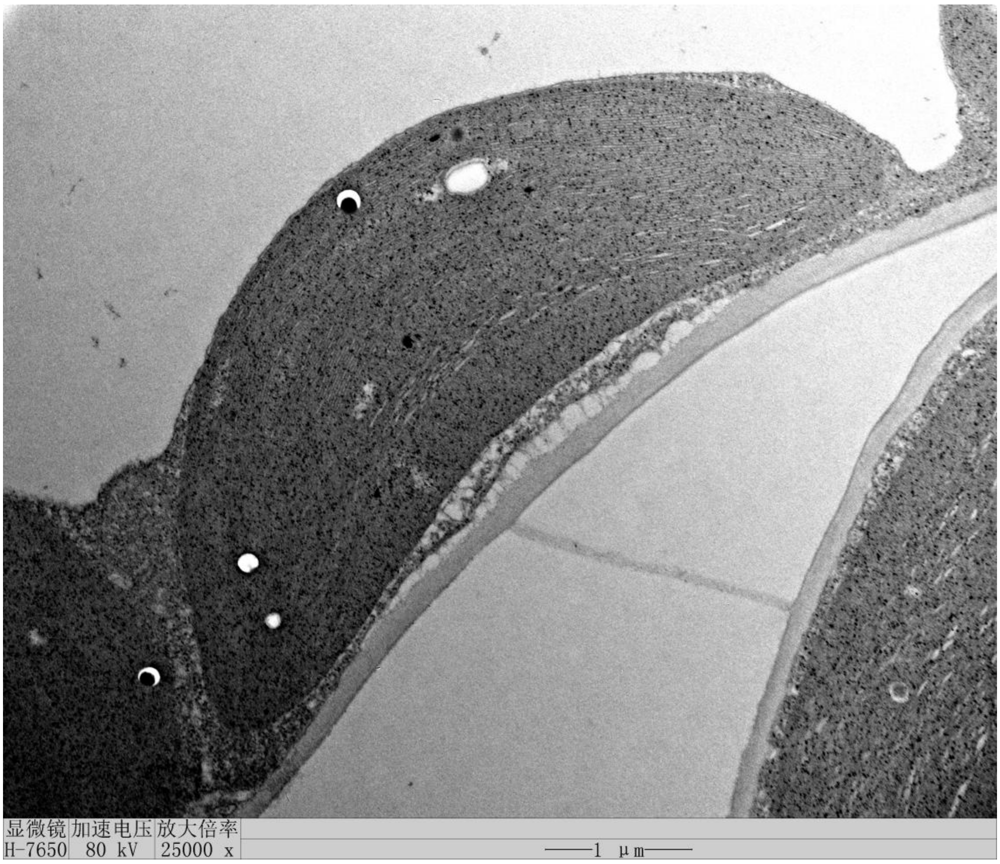

Figure 3F

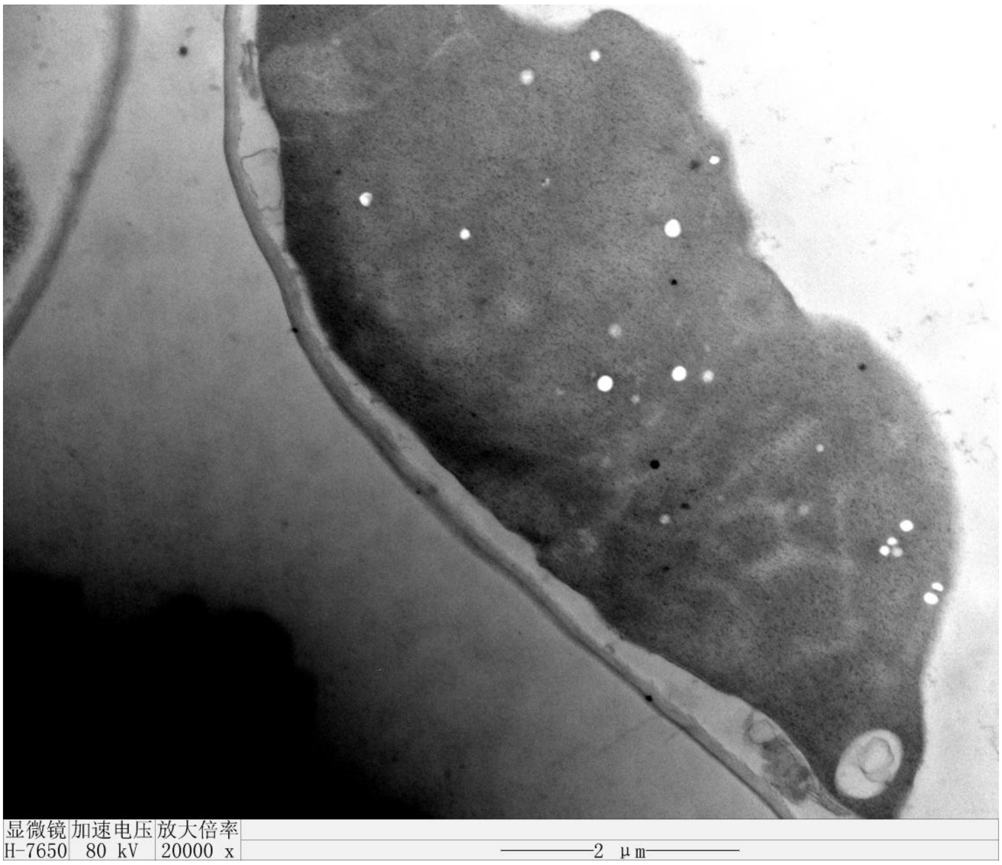

Supplement: Supplementary file 1 [file ijms-23-03340-s001.zip › supplementary files/original files/original files.pdf]
