# Supplementary material for: Chloroplast Thylakoidal Ascorbate Peroxidase, PtotAPX, Has Enhanced Resistance to Oxidative Stress in Populus tomentosa
Source: Int J Mol Sci. 2022 Mar 19;23(6):3340. doi: 10.3390/ijms23063340 (PMC8953715; doi:10.3390/ijms23063340)
Supplement: Supplementary file 1 [file ijms-23-03340-s001.zip › supplementary files/Supplementary information.pdf]

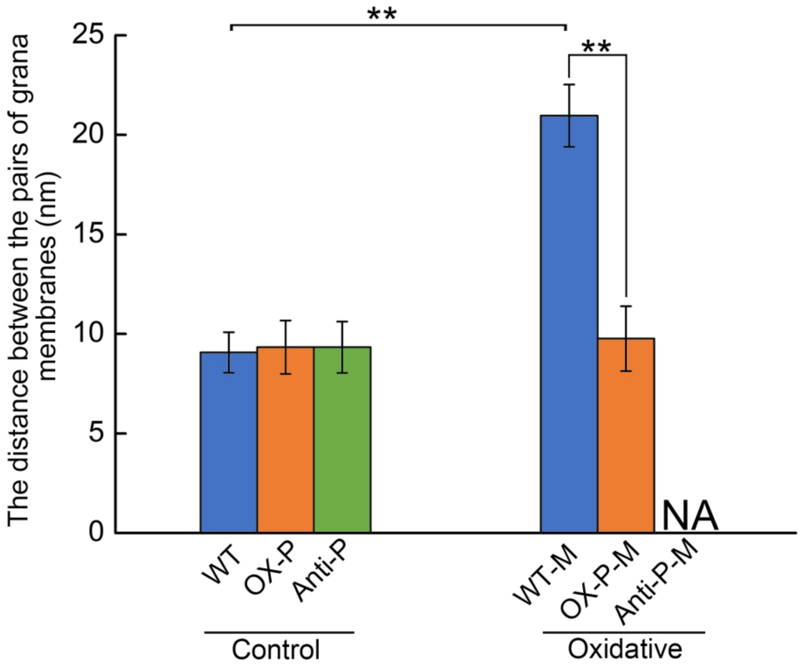

Supplementary Figure 1. The distance between the pairs of grana membrances of wild-type and transgenic plants untreated or treated with 100 μM MV.

Micrographs of sections from 3 specimens were examined. Statistical differences were determined. Values are means ± SD (n = 30). Significant differences between mean values are indicated by asterisks using Student's t-test. ** and * indicate $P < 0.01$ and $P < 0.05$, respectively.

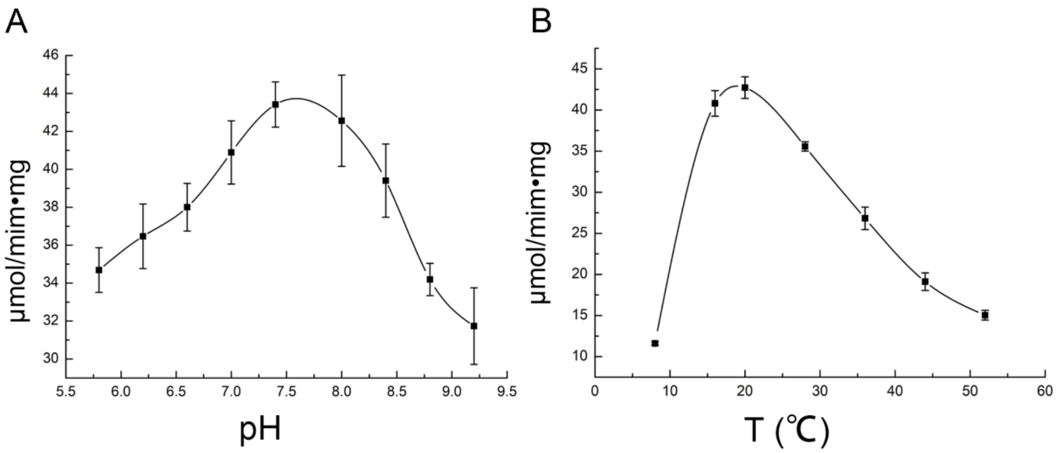

Supplementary Figure 2. The optimal pH and temperature of PtotAPX.

Supplementary Table 1. Sequences of primers used in this study.

Primers	Primer sequences（5′-3′）	Restriction sites
PtotAPX-F	ATGGCTTCTCTCAGTGGTGGTG	N/A
PtotAPX-R	CTTCCAAGTTTTATATGTGATTTC	N/A
P-PtotAPX-F	CGGGATCCTACTCTCCTTCCTCTCTCA	*BamH* I
P-PtotAPX-R	CCCAAGCTTAACTTAATTTCCAAGAAGAG	*Hind* III
qRT-actin-F	AAACTGTAATGGTCCTCCCTCCG	N/A
qRT-actin-R	GCATCATCACAATCACTCTCCGA	N/A
qRT-PtotAPX-F	CTGGAAAGAGAGAGTTGTCAG	N/A
qRT-PtotAPX-R	GTGCCAGAACAGCAATCAC	N/A
