# Supplementary figures and images for: Chloroplast Thylakoidal Ascorbate Peroxidase, PtotAPX, Has Enhanced Resistance to Oxidative Stress in Populus tomentosa
Source: Int J Mol Sci. 2022 Mar 19;23(6):3340. doi: 10.3390/ijms23063340 (PMC8953715; doi:10.3390/ijms23063340)

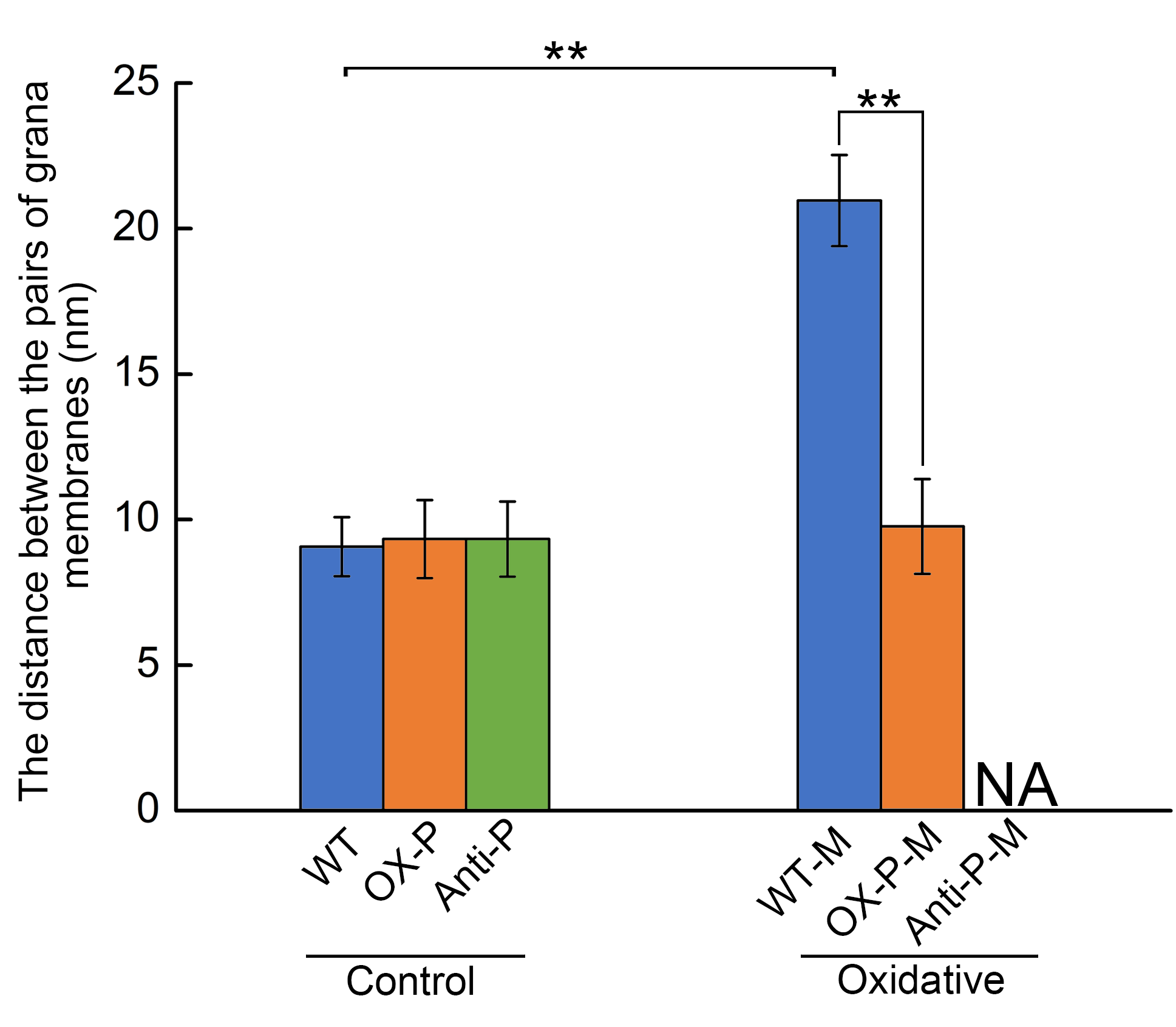

Supplement: Supplementary file 1 [file ijms-23-03340-s001.zip › supplementary files/Supplementary Figure 1.tif]

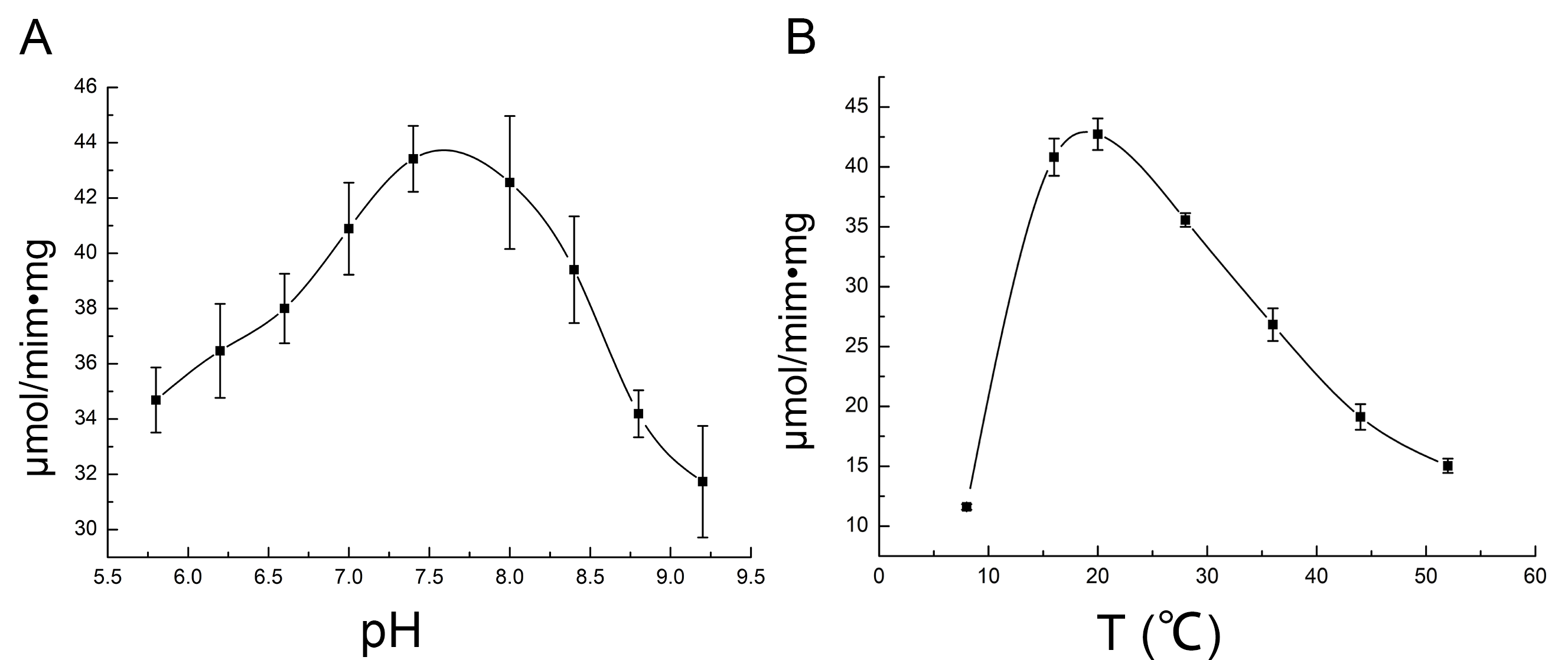

Supplement: Supplementary file 1 [file ijms-23-03340-s001.zip › supplementary files/Supplementary Figure 2.tif]
